# Targeting FGFR1 to suppress leukemogenesis in syndromic and *de novo* AML in murine models

**DOI:** 10.18632/oncotarget.10438

**Published:** 2016-07-06

**Authors:** Qing Wu, Aaron Bhole, Haiyan Qin, Judith Karp, Sami Malek, John K Cowell, Mingqiang Ren

**Affiliations:** ^1^ Cancer Center, Georgia Regents University, Augusta, GA, USA; ^2^ Sidney Kimmel Cancer Center at Johns Hopkins University, Baltimore, MD, USA; ^3^ Department of Internal Medicine, Division of Hematology and Oncology, University of Michigan, Ann Arbor, MI, USA

**Keywords:** AML, FGFR1, therapeutics, NSG-SGM3 mice, xenograft

## Abstract

Although over expression of chimeric FGFR1 kinase consistently leads to the development of AML in the rare Stem Cell Leukemia and Lymphoma syndrome, we now show that overexpression of FGFR1 is also seen in up to 20% of non-syndromic, *de novo* AML. To determine whether targeting FGFR1 in both of these AML subtypes can suppress leukemogenesis, we evaluated the effects of different FGFR1 inhibitors in a side-by-side comparison for their ability to affect *in vitro* proliferation in FGFR1 overexpressing murine and human cells lines. Three newly developed pan-FGFR inhibitors, AZD4547, BGJ398 and JNJ42756493, show a significantly improved efficacy over the more established FGFR inhibitors, PD173074 and TKI258. To examine whether targeting FGFR1 suppresses leukemogenesis in *de novo* AML *in vivo*, we created xenografts in immunocompromized mice from primary, *de novo* AML that showed > 3-fold increased expression of FGFR1. Using BGJ398, the most potent inhibitor identified in the *in vitro* studies, AML progression in these mice was significantly suppressed compared with vehicle treated animals and overall survival improved. Importantly, no difference in disease course or survival was seen in AML xenografts that did not show overexpression of FGFR1. These observations support the idea that FGFR1 is a driver oncogene in *de novo*, FGFR1-overexpressing AML and that molecularly targeted therapies using FGFR1 inhibitors may provide a valuable therapeutic regimen for all FGFR1-overexpressing AML.

## INTRODUCTION

Fibroblast growth factors (FGF) and their receptors (FGFR) play fundamental roles in many physiologic processes, including embryogenesis and adult tissue homeostasis [1–3]. Aberrant activation of FGFR1 signaling, resulting from FGFR1 amplification or mutation, has been increasingly found to be a driving factor in tumorigenesis for multiple types of cancers [4–9]. In particular, an increasing number of novel FGFR1 fusion genes have been identified by RNA-Seq analysis in different types of cancers [10, 11]. The only consistent association of FGFR1 abnormalities with cancer, however, involves the constitutive activation of a ligand-independent, chimeric FGFR1 kinase in Stem Cell Leukemia and Lymphoma syndrome (SCLL). This activation results from chromosome translocations that juxtapose the kinase domain of FGFR1 with dimerization domains from a variety of partner genes. These patients develop a myeloproliferative disease that progresses to AML in 80% of patients [12], which is classified by the WHO as myeloid and lymphoid malignancies associate with FGFR1 abnormalities [13]. Reflecting the stem cell nature of the disease, these patients may coincidentally develop T- or B-cell lymphoma [12, 14]. The current, standard treatment for SCLL has been adapted from conventional regimens developed for ALL, AML and other leukemias, but survival is still relatively poor with only ~15% of patients showing a survival of > 15 months [12].

To investigate the molecular etiology of this disease and design therapeutic strategies, we have developed mouse models for several different SCLL-derived chimeric FGFR1 kinases using transduction and transplantation of bone marrow cells [15–17]. Where it has been studied, these mice develop the same MPD, leukemias and lymphomas seen in the human disease, and genomic analyses show that consistent genetic changes in these murine leukemias are the same as those seen in the primary human disease. More recently we have developed a human cell model of SCLL through transduction of CD34+ human cord blood cells engrafted into immunocompromized mice, where again the course of the disease and the molecular genetic changes follow those seen both in primary SCLL, as well as in the murine models [16]. During the course of these studies we developed a variety of cell lines from the mouse models that overexpress FGFR1 kinase and which were shown to be sensitive, *in vitro* and *in vivo*, to treatment with ponatinib, a relatively non-specific FGFR1 kinase inhibitor [18].

Recently, several additional, more specific pan-FGFR inhibitors have been developed, such as AZD4547 [19], BGJ398 [20, 21], JNJ42756493 [22] and LY2874455 [23], in addition to multi-target inhibitors such as ponatinib (also known as AP24534) [24], TKI258 [25] and E3810 [26]. Ponatinib (Iclusig^®^) is an FDA approved drug for the treatment of chronic myeloid leukemia (CML) and Philadelphia chromosome–positive acute lymphoblastic leukemia (ALL). The other inhibitors are currently in phase I, II or III clinical trials. In general, all of these drugs have been shown to be pan-FGFR inhibitors and their ability to inhibit FGFR kinase enzyme activity has been determined using either *in vitro* based biochemical assays [19–26], or inhibition of cell proliferation using the BaF3 murine B-cell leukemia cell line with exogenous expression of different mutated FGFR genes. Several human cancer cell lines derived from solid tumors that show various FGFR mutations have also been studied using these drugs. Most of these studies, however, have only evaluated their efficacy and specificity in isolation, and their relative ability to inhibit FGFR1 kinase in the same homogeneous system has not been evaluated.

Xenografts of murine leukemia and lymphoma cell lines in mice have allowed evaluation of the ability of various FGFR1 inhibitors to suppress leukemia progression *in vivo*, but possibly do not represent the heterogeneity seen in primary human leukemias. To overcome this limitation, we have developed a xenograft system for human AML using a new, immunocompromised transgenic NSG mouse strain (NOD.Cg-Prkdc^scid^-Il2rg^tm1WjlTg^) endogenously expressing human SCF, GM-CSF, and IL-3 cytokines (NSG-SGM3) [27] using approaches described previously [18]. A sub set of non-syndromic, *de novo* human AMLs that have been shown to overexpress FGFR1 have been successfully engrafted into these NSG-SGM3 mice. In this study, we have used both FGFR1-dependent murine leukemia cell lines carrying different chimeric FGFR1 fusion kinases as well as FGFR1-dependent lung and breast cancer lines with amplification of FGFR1, to compare the ability of 5 different pan-FGFR inhibitors to suppress FGFR1 activation and subsequent leukemogenesis, We show that BGJ398 is the most efficient inhibitor based on *in vitro* cell growth inhibition and apoptosis assays. When xenografts of human AML cells overexpressing FGFR1 were treated with BGJ398, there was a significant inhibition of leukemogenesis, suggesting targeting FGFR1 in this subset of AML may be an effective therapy.

## RESULTS

### Comparison of the efficacy and specificity of FGFR inhibitors in solid tumor cell lines with FGFR1 amplification

**Figure 1:**
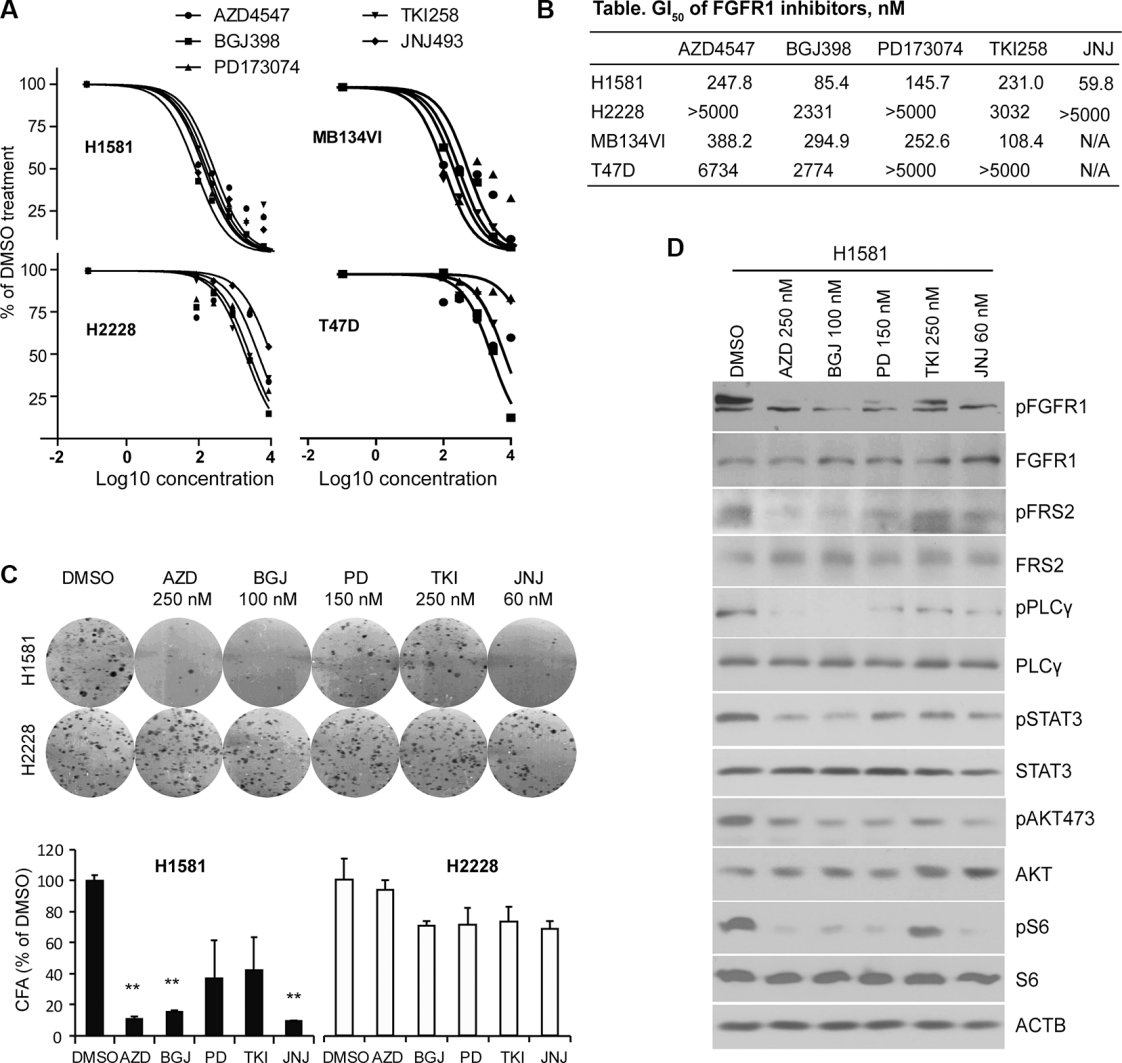
GI_50_ levels for lung and breast cancer cells treated with FGFR inhibitors (**A**) Growth inhibition curves, performed in quadruplicate, for human lung cancer (H1581 and H2228) and breast cancer (MB-134VI and T47D) cells treated with 5 different pan-FGFR inhibitors. H1581 and MB-134VI cells carry FGFR1 amplification. To avoid confusing congestion within the figure by including all four individual data marks and related standard deviations, only mean values are plotted, although in all cases SD values were < 10% of the meanvalue. (**B**) Summary of the GI_50_ concentrations for each of FGFR inhibitors. All cell lines were treated with the individual FGFR inhibitors at 0, 300, 1000, 3000, 10,000 nM for 72 h. GI_50_ values were calculated using GraphPad Prism 5 software. (**C**) The FGFR inhibitors remarkably suppress colony formation for H1581 cells but not H2228 cells. Data are presented as mean ± SD. * = *p* < 0.05, ** = *p* <0.01 compared with the vehicle-treated control. (**D**) Western blot analysis (at least in duplicate for all proteins) shows that the phosphorylation levels of FGFR1 and its downstream signaling targets were dramatically reduced by all FGFR inhibitors in H1581 cells.

Several novel pan-FGFR inhibitors have been recently developed [19–26], which have shown promise in either preclinical or clinical trials for solid tumors overexpressing FGFR1 [9, 10, 28, 29]. Among these, inhibitors AZD4547, BGJ398 and JNJ42756493, were selected because they have been shown to effectively inhibit FGFR1 kinase in biochemical assays [19–22]. However, since each inhibitor has been investigated in isolation, in different model systems, it has not been possible to determine the relative efficacy and specificity of these drugs in inhibiting FGFR1 activity and related phenotypes. To evaluate the relative effect of the FGFR inhibitors we first used two cell lines derived from solid tumors, the H1581 large-cell lung carcinoma cell line and the human MDA-MB-134VI breast cancer cell line, both of which overexpress FGFR1 and have been shown to be sensitive to at least one of these FGFR inhibitors [8, 9]. As negative controls, we also included the H2228 human lung cancer and T47D human breast cancer cell lines which show low or no expression of FGFR1-4 [8, 9]. To evaluate the concentration for 50% maximal inhibition of cell proliferation (GI_50_) in these cells, we treated them with individual FGFR inhibitors for 72 h at concentrations of 0, 100, 300, 1000, 3000 and 10000 nM. All of the FGFR inhibitors (< 400 nM) remarkably inhibited cell proliferation of H1581 and MB134VI cells, but did not inhibit H2228 or T47D cells (Figure 1A). The GI_50_ values were calculated as described in Figure 1B. To further compare cell growth inhibition of these FGFR inhibitors, we next performed colony formation assays (CFA), where the cells were exposed to drugs (at GI_50_) for only 24h and then allowed to grow for 12 days in drug-free medium (see Materials and Methods). CFA analysis clearly shows that AZD4547, BGJ398 and JNJ42756493 were more efficient in inhibiting colony formation for H1581 cells compared with PD173074 and TKI258 (Figure 1C). H2228 cells were not affected. Consistent with the biological effect, AZD4547, BGJ398 and JNJ42756493 were more effective in suppressing FGFR1 phosphoactivation in H1581 cells than the other two drugs (Figure 1D). In addition, phosphorylation levels of downstream components of FGFR1 signaling, such as FRS2, PLCγ, STAT3, pS6 and pAKT473 (Figure 1D), were also suppressed. However, we did not observed significant changes in pAKT308 levels (not shown). Overall, the pan-FGFR inhibitors BGJ398, AZD4547 and JNJ42756493 efficiently inhibited proliferation of cells showing FGFR1 amplification.

### Effect of FGFR inhibitors on cell proliferation in FGFR1-dependent leukemia cell lines

**Figure 2:**
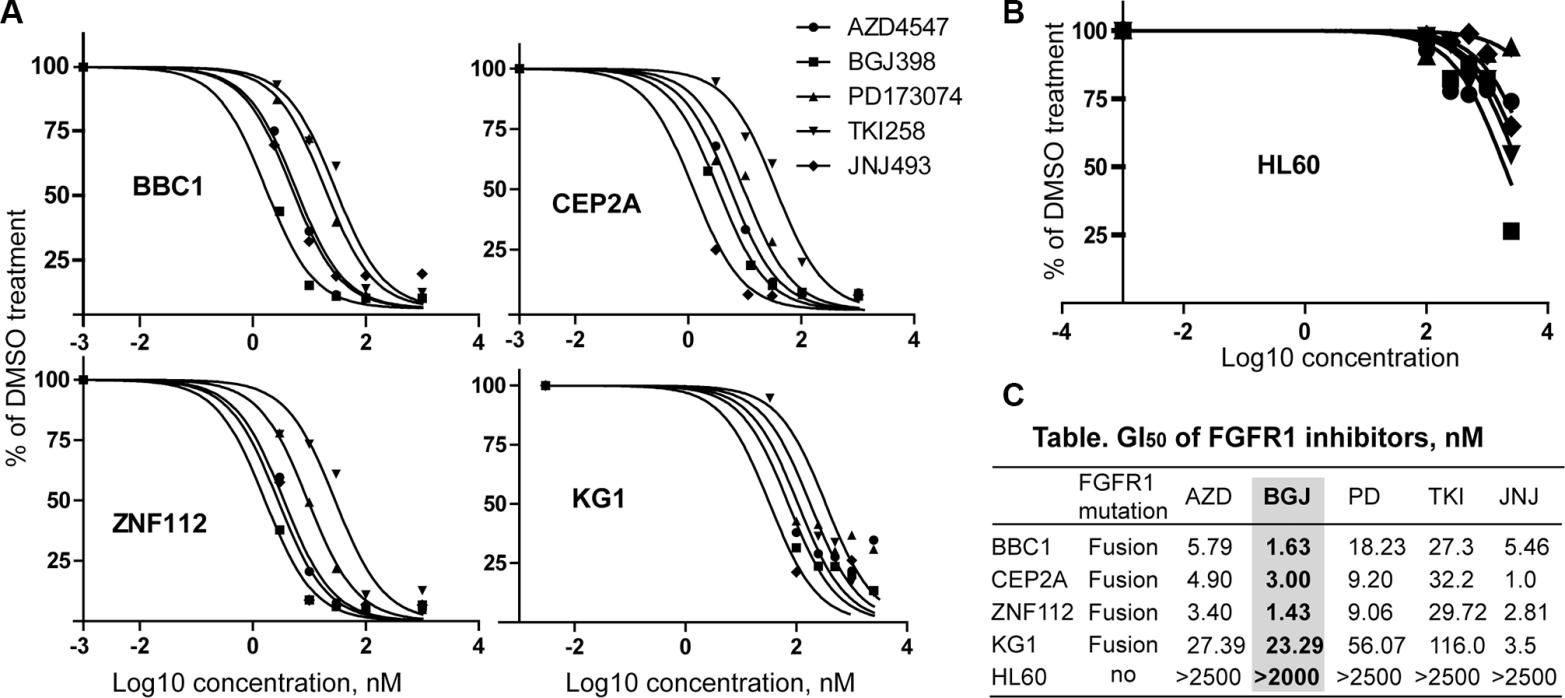
GI_50_ of leukemic cell lines for FGFR inhibitors (**A**) Growth inhibition curves (preformed in quadruplicate) for murine leukemic cell lines (BBC1, CEP2A, and ZNF112) and the human KG1 AML cell line following treatment with 5 different pan-FGFR inhibitors. To avoid confusing congestion within the figure by including all four individual data marks and related standard deviations, only mean values are plotted, although in all cases SD values were < 10% of the mean value. (**B**) Growth inhibition curve for HL60 human acute myeloid leukemia cells, which do not express FGFR1. (**C**) Summary of GI_50_ values for each of the FGFR inhibitors. GI_50_ value (the concentration for 50% of maximal inhibition of cell proliferation) was calculated using GraphPad Prism 5 software. BGJ398 is highlighted to show consistent inhibition across all FGFR1 expressing cell lines.

Through our efforts in developing mouse models for leukemogenesis driven by various chimeric FGFR1 kinases, we developed a series of cell lines that express three different chimeric FGFR1 fusion genes; ZNF112 expresses ZMYM2-FGFR1 [30], BBC1 and BBC2 express BCR-FGFR1 [17] and CEP2A and CEP5A express CNTRL-FGFR1 [18]. These leukemic cell lines are all dependent on activation of FGFR1 signaling for survival, since treatment with ponatinib, for example, leads to growth suppression [18]. The human KG1 cell line [31], which expresses a chimeric FGFR1OP2-FGFR1 gene, was also included in the analysis. These lines were used to evaluate the efficiency of FGFR1 inhibitors to suppress leukemic cell growth *in vitro*. For comparison, we also included the PD173074 and TKI258 FGFR inhibitors, which have been extensively used in *in vitro* and *in vivo* studies [9, 32, 33]. To measure GI_50_ values, we treated cells with individual FGFR inhibitors at concentrations ranging between 0 to 1000 nM. Since these leukemic cells grow faster than the solid tumor cells, they were treated for 48 h. Human HL60 AML cells, that do not express FGFR1 [18], were used as a negative control. As shown in Figure 2A, all of the FGFR1 overexpressing leukemic cells were sensitive to the FGFR inhibitors but HL60 cells were not (Figure 2B). Based on the GI_50_ values, as seen in the study of solid tumor cell lines, AZD4547, BGJ398 and JNJ42756493 proved much more efficient (lower concentration of drug) in inhibiting cell growth of leukemic cell lines showing elevated levels of FGFR1 expression, compared with PD173074 or TKI258 (Figure 2C).

**Figure 3:**
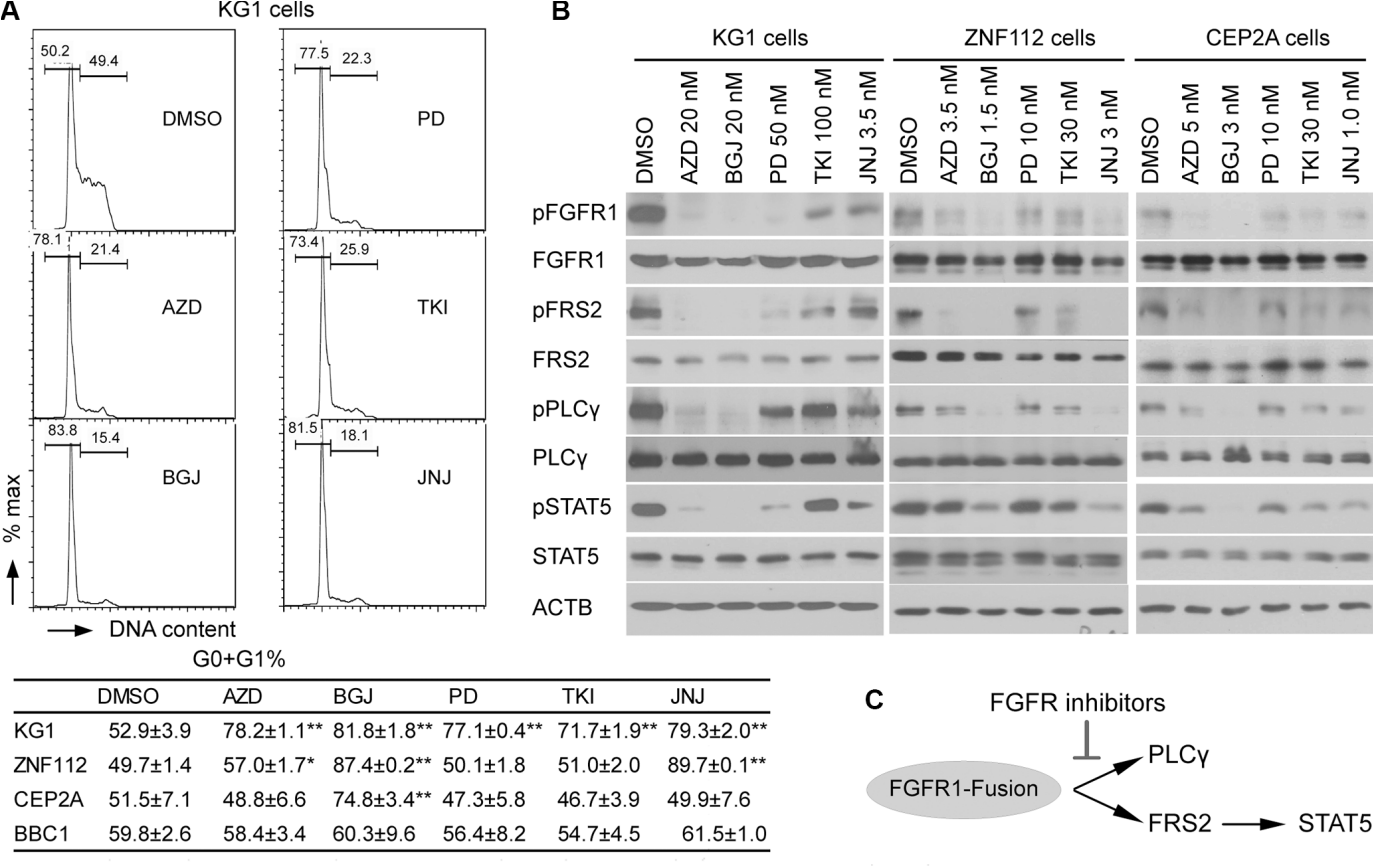
Cell growth inhibition induced by FGFR inhibitors is consistent with downregulation of FGFR1 phosphorylation (**A**) A representative cell cycle analysis from KG1 cells treated with the five FGFR inhibitors (in duplicate) for 48 h at the GI_50_ concentration is shown (upper panel). The percentage changes in the G0+G1 population of cells for the different cell lines treated with different drugs are shown below. Data were analyzed using the Student's *t*-test (two-tailed). Data are presented as mean ± SD. * = *p* < 0.05, ** = *p* < 0.01 compared with the vehicle-treated control. (**B**) Western blot analyses show the relative inhibition of phosphorylated FGFR1, as well as downstream FGFR1 target proteins in leukemic cells following treatment with different FGFR inhibitors. The leukemic cells were treated for 12 h at the corresponding GI_50_ concentration. All western blot analyses were at least duplicated. (**C**) Scheme of effects of FGFR1 inhibitors on downstream effectors of FGFR1 signaling, PLCγ and FRS2-STAT5.

We next determined whether the inhibition of cell growth was due to cell cycle inhibition. The same leukemia cells, therefore, were treated individually with each inhibitor at its GI_50_ concentration. After 48 h treatment, the cell cycle was analyzed using flow cytometry. As shown in Figure 3A, BGJ398 leads to the most efficient increase in cell cycle arrest in all cells tested. JNJ42756493 also induced significant cell cycle arrest in KG1 and ZNF112 cell lines. To investigate whether cell growth inhibition was associated with inactivation of FGFR1 signaling, we treated the cells with these inhibitors at their GI_50_ for 12 h and then used western blotting to evaluate the levels of activated proteins downstream in the FGFR1 signaling pathway. Phosphorylation levels of FGFR1 were consistently suppressed by AZD4547 and BGJ398 in these cell lines (Figure 3B) and, as a result, reduced phosphorylation levels of FRS2, STAT5 and PLCγ were observed. Overall, based on the GI_50_, and cell growth inhibition as well as downstream molecular effects, BGJ398, AZD4547 and JNJ42756493 are relatively more effective and specific in targeting the FGFR1 signaling pathway in leukemia cell lines carrying FGFR1 fusion kinases.

### BGJ398 suppresses leukemogenesis in FGFR1 overexpressing primary AML in a mouse xenograft model

**Figure 4:**
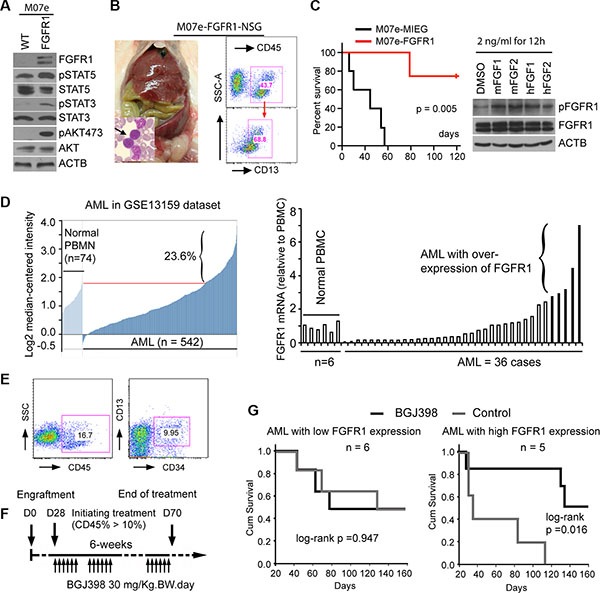
Targeting FGFR1 in *de novo* AML overexpressing FGFR1 with BGJ398 suppresses leukemogenesis in a mouse xenograft model (**A**) Western blot analysis following overexpression of wild type FGFR1 in M07e cells shows activation of STAT5, STAT3, and AKT compared with actin (ACTB) levels. (**B**) M07e-FGFR1 or M07e-MIEG3 (control) cells were transplanted into NSG mice (2 × 10^6^ cells per mouse). After 2 months the M07e-FGFR1 injected mice show enlarged spleens and high blast count in the peripheral blood (arrowed in the insert). Flow analysis shows a human CD45+CD13+ immunophenotype from a diseased mouse bone marrow (right). (**C**) M07e-FGFR1 mice (*n* = 5) have a significantly shorter survival time (left) than the M07e-MIEG3 mice (*n* = 4). One of the M07e-MIEG3 mice died unrelated to leukemia. Treatment of H520, FGFR1-overexpressing lung cancer cells with mouse FGFR1/2 ligands (mFGFR1/2) or human FGF1/2 ligands (hFGF1/2) at a final concentration of 2 ng/ml for 12 h, leads to activation of human FGFR1 (right). (**D**) Analysis of FGFR1 gene expression levels in GEO data set GSE13159 reveals that > 20% of AML show increased expression levels (left). Quantitative RT-PCR analysis of primary AML samples xenografted in NSG-SGM3 mice shows a subpopulation (*n* = 5) of AML patients that express FGFR1 at least 3 times higher than that in peripheral blood mononuclear cells (PBMC) derived from normal healthy individuals. (**E**) Flow cytometry analysis from a representative AML-engrafted mouse (left panel), shows ~16% CD45+ human leukocytes in peripheral blood. (**F**) Schematic presentation of the treatment regimen used in this protocol. (**G**) Kaplan-Meier survival analysis shows no significant improvement in survival in the cohort engrafted with AML cells showing low FGFR1 expression (*n* = 6), but there is a significant improvement in survival in the cohort showing increased expression of FGFR1 (*n* = 5).

Several studies have demonstrated that increased FGFR1 activity, as a result of FGFR1 fusion kinases, drive leukemia/lymphoma development in mouse models. However, whether overexpression of full-length FGFR1 also promotes tumorigenesis in myeloid cells is not known. A suggestion that this may be the case comes from the observation that Trisomy/polysomy for chromosome 8 (the location of the FGFR1 gene) is the most frequent (~10%) chromosome change in AML [34]. This phenomenon led us to investigate whether overexpression of FGFR1 plays a pathological role in leukemogenesis. Using the classic IL-3-dependent, pre-B-cell BaF3 (murine) cell line model, we showed that overexpression of FGFR1 transformed these cells into IL-3-independence. Extending this studies into the non-tumorigenic human IL3-dependent M07e (Figure 4A) cells [35], overexpression of FGFR1 also led to IL3-independent growth *in vitro* and, when xenografted into NSG immunodeficient mice, led to the development of leukemia, unlike mice engrafted with M07e cells carrying the empty MIEG3 vector (Figure 4B and 4C). Thus, as with cell lines expressing the constitutively active chimeric FGFR1 genes, overexpression of full-length FGFR1 also promotes leukemic transformation. Analysis of downstream pathways showed that constitutive expression of FGFR1 in M07e cells activated typical FGFR1 targets such as STAT3, AKT and STAT5 (Figure 4A).

The observation that increased FGFR1 activity, whether through overexpression or upregulation through chromosome translocations is implicated with leukemia development, led us to investigate whether FGFR overexpression is a frequent event in *de novo* AML. Analysis of gene expression data from public databases (GSE13159), showed that approximately 20% of AML (Figure 4D) carry increased FGFR1 transcription levels compared with normal peripheral blood mononuclear cells (PBMC) and a significant proportion express even lower levels. To investigate whether these FGFR1 overexpressing AML are also sensitive to FGFR1 inhibitors, we designed a strategy to generate xenografts of FGFR1-overexpressing AML. To identify these specific AML, we systematically performed RT-PCR analysis of primary human AML samples that had been cryogenically preserved in tumor repositories from Georgia Regents University and the University of Michigan. Of the 58 samples analysed, five showed at least a 3-fold increased expression of FGFR1 compared with normal peripheral blood leukocytes (Figure 4D). To determine whether human FGFR1 will be activated in this mouse system we demonstrated, that murine FGFR1 ligands, such as FGF1 and FGF2 can activate human FGFR1 using the human H520 lung cancer cell line that overexpresses FGFR1, (Figure 4C). The five de novo FGFR1^high^ AML were then xenografted into NSG-SGM3 mice, which all developed leukemia within 2–3 months as shown by increased blast counts in the peripheral blood. The immunophenotypes of these xenografts were CD45^+^CD34^+/−^(Figure 4E). As controls, we also engrafted six primary AML that did not overexpress FGFR1. Unfortunately, because of the limited amount of material, we were only able to engraft two mice for each patient sample, and so could not evaluate all of the FGFR1 inhibitors *in vivo*. We therefore selected BGJ398, which was the best performing drug *in vitro*, to conduct an *in vivo* study to determine whether targeting FGFR1 in the overexpressing xenografts could suppress leukemia development using the strategy shown in Figure 4F. The mice engrafted with primary AML cells from the same patient were randomized to the treatment (BGJ398, 30 mg/kg-BW) or vehicle (control) groups, respectively once the number of human CD45+ cells exceeded 5% in the peripheral blood. The dose of BGJ398 was selected based on a previous report [21]. All mice were treated orally using a gavage needle either with BGJ398 or vehicle control daily. All treatments were performed for 5 days per week for 6 weeks. As shown in Figure 4G, treatment with BGJ398 significantly (*P* = 0.016) prolonged survival in the cohort of mice xenografted with leukemias over-expressing FGFR1, but not in the AML group showing low expression of FGFR1 (Figure 4G). Thus it appears that FGFR1 is a driver event in these *de novo* AML and targeting its function could be a rational approach for therapy in this subclass of AML.

## DISCUSSION

AML is a heterogeneous disease and it is now evident that there are many different molecular etiologies, with currently 54 cytogenetic subtypes [34] most of which occur with a frequency of less than 1%. It is likely, therefore, that molecular targeting of AML may have to consider one sub type at a time, no matter how rare the disease. Clearly, even though the genetics of these subgroups will be important in the overall strategy for treating AML in the future, identification of more common genetic events that occur across the groups, present more common targets that may streamline future therapeutic approaches. We now show that a relatively large proportion of AML overexpress FGFR1, and that targeting this kinase may prove effective for this sub group.

The initial observation of FGFR1 overexpression came from a bioinformatics study of gene expression data available in public databases, where FGFR1 overexpression was seen in > 20% of AML, although with no verification of the primary data. In our study using qRT-PCR, however, the incidence was nearer ~10%, albeit in a study involving only 58 cases. Nonetheless, this frequency exceeds many other genetic events seen in AML. Overexpression of FGFR1 is also the driver of SCLL, which is similarly sensitive to anti-FGFR1 therapies [18, 36–38]. The presence of FGFR1 overexpression in these AML, however, was confirmed using quantitative PCR, unlike those in the databases. Identification of AML that could potentially benefit from anti-FGFR1 treatment based on expression levels alone, however, may be an underestimate, since there may be mutational activation or other mechanisms of enhanced protein activation that are independent of the expression level. Stratifying patients for an anti-FGFR1 therapy, therefore, may require development of a more comprehensive survey of FGFR1 activation status.

The opportunity to consider targeting FGFR1 in clinical trials for AML is supported by the progressive discovery of inhibitors with greater specificity. Early FGFR1 inhibitors, such as ponatinib, were developed as drugs that targeted other kinases but were subsequently shown to also affect FGFR1 activation [24]. We have previously shown that the multikinase inhibitor ponatinib can suppress the development of myeloid and lymphoid malignancies associated with FGFR1 abnormalities both *in vitro* and *in vivo* [18]. Consistent with our preclinical studies, ponatinib has now been used clinically to treat an SCLL-related AML with the BCR-FGFR1 chimeric kinase which showed a good outcome. Twelve weeks after starting ponatinib, the bone marrow in this patient showed a complete morphologic remission [36]. In our previous study, ponatinib at 40 mg/kg body weight significantly decreased the body weight of the mice [18]. Indeed, > 8% of patients treated with ponatinib developed cardiovascular, cerebrovascular, and peripheral vascular thrombosis, including fatal myocardial infarction and stroke [39]. Unlike multikinase inhibitors, such as ponatinib, TKI258 and E3810, however, the BGJ398, JNJ42756493 and AZD4547 inhibitors appeared to specifically inhibit FGFR kinases and were shown to be very potent in suppressing growth in the FGFR1-dependent leukemia cells in our study. More importantly, we did not observe any obvious side effects in mice treated with BGJ398. Thus, specific FGFR inhibitors with narrow toxicity profiles may prove especially important in those FGFR-dependent malignancies.

## MATERIALS AND METHODS

### FGFR1 inhibitors

AZD4547 and BGJ398 were purchased from the ChemieTek, JNJ42756493 from Active Biochem, TKI258 from LC Laboratories and PD173074 from Cayman Chemical. All drugs were diluted in DMSO, aliquoted and stored as 10 mM stocks at −80°C.

### Cell culture and proliferation assays

Murine leukemia cell lines were isolated from mice that had developed leukemias carrying different chimeric FGFR1 genes as described previously [18] and their immunophenotype was confirmed using standard flow cytometry analysis. The KG1 cell line was shown to carry the FGFR1OP2-FGFR1 rearrangement demonstrating the identity of this cell line. Human lung H1581 and H2228 and breast MDA-MB-134VI and T47D cancer cells were purchased from ATCC (passage number < 15). All cell lines were cultured in RPMI (Invitrogen) with 5% FBS (Hyclone), at 37°C in 10% CO_2_. For drug treatments, 40,000 leukemia cells/well or 5,000 solid tumor cells/well were seeded in 96-well plates and incubated overnight, then treated with the either DMSO (control) or the drugs indicated in the results section at concentrations defined by the experiments. Cell viability was determined using Cell Titer-Glo luminescence cell viability kits (Promega) and a SpectraMax^®^ M5e (Molecular Probe) luminescence plate reader [18].

### Cell cycle analysis

Cell cycle analysis was performed using standard flow cytometry procedures following propidium iodide staining as described [18].

### Colony formation assay

Cells (500–1,000/well) were seeded in 6-well plates in DMEM medium plus 5% FBS, allowed to grow for 24 h before treatment with drugs or vehicles (controls). After 24 h treatment, the wells were washed twice with 37°C warmed PBS and then growth media was added. The media were changed every 3 days. As the colonies became visible (usually after 12 days), cells were fixed with methanol, stained with Giemsa (1:10 in distilled water) and counted as described [16].

### Western blot analyses

Whole-cell lysates (30 μg) were separated using SDS-PAGE and immunoblotted with specific antibodies. Anti-phospho-FGFR1 antibody was purchased from Abcam, anti-FGFR1 antibody (Santa Cruz Biotechnology). The other antibodies have been described previously [18].

### Patient samples

For the *in vivo* xenograft studies, all viably frozen bone marrow cells were collected under informed consent protocols approved by the respective organizations to use residual material for research purposes in accordance with the Declaration of Helsinki. AML cells were engrafted into immunocompromised NSG-SGM3 mice via tail vein injection as described previously (15–16). An aliquot of the frozen AML cells were also used for DNA and RNA extraction using Trizol reagent (Life Technologies) for molecular analysis.

### Animals and drug regimens

NSG-SGM3 mice were originally obtained from the Jackson Laboratory and maintained as a breeding colony at GRU. All experiments were conducted under GRU IACUC approved protocols. Female, 6–8 week old mice were used in all xenograft experiments. Mice were engrafted with 1–2 × 10^6^ AML cells via tail vein injection. Each primary AML sample was engrafted into two mice. All engrafted NSG-SGM3 mice were monitored for leukemia development using flow cytometry and when they showed > 5% human CD45+ cells in their peripheral blood drug treatment was initiated. The mice engrafted with AML from the same patient were randomized to either the treatment group (BGJ398, 30 mg/kg BW) or vehicle control group, respectively. The dose of BGJ398 was selected based on a previous report [21]. All mice were treated with either BGJ398, or vehicle control (PEG300:acetic buffer = 1:1), orally using a gavage needle once per day. All treatments are performed for 5 days per week for 6 weeks.

### Statistical analysis

Survival accumulation was estimated by Kaplan- Meier analysis and the log-rank (Mantel-Cox) test. The *t*-test was used throughout in calculations between test and control groups. Statistical analysis was performed using SPSS software (SPSS Inc. Chicago, Il).
